# Attitudes towards technology supported rheumatoid arthritis care: investigating patient- and clinician-perceived opportunities and barriers

**DOI:** 10.1093/rap/rkad089

**Published:** 2023-10-26

**Authors:** Amy MacBrayne, Paul Curzon, Hamit Soyel, William Marsh, Norman Fenton, Costantino Pitzalis, Frances Humby

**Affiliations:** Experimental Medicine and Rheumatology, William Harvey Research Institute, Queen Mary University of London, London, UK; School of Electronic Engineering and Computer Science, Queen Mary University of London, London, UK; School of Electronic Engineering and Computer Science, Queen Mary University of London, London, UK; School of Electronic Engineering and Computer Science, Queen Mary University of London, London, UK; School of Electronic Engineering and Computer Science, Queen Mary University of London, London, UK; Experimental Medicine and Rheumatology, William Harvey Research Institute, Queen Mary University of London, London, UK; Experimental Medicine and Rheumatology, William Harvey Research Institute, Queen Mary University of London, London, UK

**Keywords:** telemedicine, mHealth, rheumatoid arthritis, digital technology in medicine, questionnaire

## Abstract

**Objectives:**

Globally, demand outstrips capacity in rheumatology services, making Mobile Health (mHealth) attractive, with the potential to improve access, empower patient self-management and save costs. Existing mHealth interventions have poor uptake by end users. This study was designed to understand existing challenges, opportunities and barriers for computer technology in the RA care pathway.

**Methods:**

People with RA were recruited from Barts Health NHS Trust rheumatology clinics to complete paper questionnaires and clinicians were recruited from a variety of centres in the UK to complete an online questionnaire. Data collected included demographics, current technology use, challenges managing RA, RA medications and monitoring, clinic appointments, opportunities for technology and barriers to technology.

**Results:**

A total of 109 patient and 41 clinician questionnaires were completed. A total of 83.5% of patients and 93.5% of clinicians use smartphones daily. However, only 25% had ever used an arthritis app and only 5% had persisted with one. Both groups identified managing pain, flares and RA medications as areas of existing need. Access to care, medication support and disease education were mutually agreeable opportunities; however, discrepancies existed between groups with clinicians prioritizing education over access, likely due to concerns of data overwhelm (80.6% considered this a barrier).

**Conclusions:**

In spite of high technology use and willingness from both sides, our cohort did not utilize technology to support care, suggesting inadequacies in the existing software. The lack of an objective biomarker for RA disease activity, existing challenges in the healthcare system and the need for integration with existing technical systems were identified as the greatest barriers.

**Trial registration:**

Registered on the Clinical Research Network registry (IRAS ID: 264690).

Key messages

- Both patients and clinicians are amenable to using mHealth but do not use existing technology.
- Mutually agreed opportunities for mHealth include support with pain, flares and medication management.
- Lack of a reliable biomarker for RA activity means that remote monitoring solutions must be multifaceted.

## Introduction

RA is a chronic erosive inflammatory arthritis with a prevalence of ≈1% [1]. The advent of advanced therapeutics and a treat-to-target paradigm have significantly improved outcomes. However, this requires intensive monitoring by a rheumatologist [2] and globally demand outstrips capacity, making mobile health (mHealth) solutions an attractive prospect with the potential to improve access, empower patients to self-manage and reduce costs. Global smartphone penetration was estimated to be 83.3% in 2022 [3]. The potential for mobile technology to transform the delivery of health services globally is well recognized [4], with the coronavirus disease 2019 (COVID-19) pandemic accelerating this need with the rapid deployment of telehealth clinics [5]. However, telehealth is limited by the inability to examine the patient, and recent data support a strong preference for face-to-face (f2f) consultations by both rheumatology patients and clinicians [6]. Some of the gaps may be filled by mHealth: using validated outcome measures [7–10], visualization of painful/swollen joints on body maps [11, 12], innovative data collection using integrated smartphone biosensors [13–15] and asynchronous communication [16, 17], enabling more regular input of data on disease activity between standard f2f reviews [18].

Data suggest that while RA patients in European cohorts have high levels of smartphone usage (82.2–91.2%), and are eager to use mHealth, mHealth technologies are used by only 4.1–8% of patients [19, 20]. However, patients reported that an app could help them to self-manage their disease if it was tailored to their needs and co-developed with health professionals [21]. Physician recommendation has been found to significantly influence patients’ decisions to engage with health technologies [22].

Thus, while mHealth is a rapidly growing field, uptake by end users is variable and factors influencing this are not clearly understood. Furthermore, software developers often design without properly understanding the needs of the ultimate users, solving non-existent problems [23]. Recently, the EULAR published its points to consider for remote care in rheumatic and musculoskeletal diseases (RMDs), specifying that remote monitoring interventions ‘should be developed in collaboration with all stakeholders including the healthcare team, caregivers and people with RMDs’ [24].

Therefore, the aim of this study was to understand existing challenges for patients and clinicians managing RA, in order to delineate both explicit and implicitly identified opportunities and barriers for technology supported RA care pathways among target end users (i.e. RA patients and clinicians), based on the principles of user-centred design [23]. Furthermore, this study sought to understand rheumatologists’ attitudes towards disease activity assessment in order to design an acceptable and reliable remote monitoring tool.

## Methods

### Questionnaire design

The patient questionnaire, focused on technology in RA care, was developed through two consecutive RA patient and public involvement (PPI) group discussions (six patients), in addition to a review of the RA literature [25] and researchers’ expertise. Researchers included a rheumatologist (AMB) and human–computer interface scientists (PC, HS). Questions were designed to be broad-based, addressing the challenges of managing RA day to day to capture the areas of greatest need for technological support, in addition to addressing patients’ and clinicians’ explicit concerns about using health technology. Draft questionnaires were shared with members of the PPI group, who gave direct feedback on the structure, relevance and comprehension of the content, suggesting appropriate rephrasing. A clinician from the research team (FH), not directly involved in the questionnaire development, piloted and provided feedback on the clinician questionnaire. Questionnaires used a variety of quantitative responses, including tick-box selection, ranking options and Likert scales, in order to maximize the quality and relevance of the data collected. Questions were structured around demographic information, digital technology use, understanding living with RA, RA medications and monitoring, clinic appointments, opportunities for technology and barriers to technology. Clinician questionnaires had an additional section addressing disease activity assessment. Questionnaires are available for review in the supplementary material (available at *Rheumatology Advances in Practice* online) or https://improvinglifewithra.wordpress.com/.

### Participants

RA patients >18 years old, able to comprehend and consent to the questionnaire, were recruited from Barts Arthritis Centre, Barts Health NHS Trust, while awaiting routine rheumatology clinic appointments from October 2019 to March 2020. Paper surveys were completed while waiting or returned by post. Doctors, nurses and allied health professionals specialising in rheumatology care for ≥1 year were recruited via a series of mailing lists from multiple centres across London and southeast England. Clinician questionnaires were completed online via SurveyMonkey from November 2019 to February 2020.

Return of a completed patient or clinician questionnaire was taken as implicit consent to participate in the questionnaire arm of the study. The study was registered (CMPS ID 43816) and ethical approval was obtained through the Bloomsbury Research and Ethics Committee (19/LO/1345).

### Statistical analysis

Questionnaire data were analysed with descriptive statistics using SPSS version 26 (IBM, Armonk, NY, USA). For each variable, descriptive statistics (number, percentage, mean and s.d.) were calculated.

## Results

### Demographics

#### People with RA

Of 114 questionnaires collected, 5 were excluded due to insufficient information to verify the participant’s identity, leaving 109 suitable for analysis. The mean age was 56.1 years (s.d. 15.1), with the majority of participants being female (81.6%). A total of 45% of participants were degree educated, but only 36.6% were employed (24.8% not working due to their health). The mean 28-joint DAS (DAS-28) was 3.6 (s.d. 1.78), although 53.2% of patients did not have a DAS at their last clinical review (Table 1).

**Table 1.** Patient and clinician demographics

Characteristics	Patient questionnaire (*n* = 109)	Clinician questionnaire (*n* = 41)
Age, years, *n* (%)		
18–34	10 (9.2)	10 (24.9)
35–54	38 (34.9)	27 (63.9)
55–74	52 (47.7)	4 (9.6)
>75	9 (8.3)	0 (0)
Mean (s.d.)	56.1 (15.1)	–
Female, *n* (%)	89 (81.6)	26 (65.0)
Ethnicity, *n* (%)		
Asian/Asian British	43 (39.8)	–
Black/Black British	9 (8.3)	–
White British	41 (38)	–
White other	8 (7.4)	–
Other	9 (8.3)	–
Education level, *n* (%)		
Degree/postgraduate	45 (41.7)	–
A-level/equivalent	11 (10.2)	–
GCSE/equivalent	24 (22.2)	–
Other qualification	7 (6.5)	–
No qualification	18 (16.7)	–
Employment status, *n* (%)		
Yes	41 (36.6)	–
No, retired, other	17 (15.6), 42 (38.5), 9 (8.3)	–
Not working due to health	27 (24.8)	–
Clinician role, *n* (%)		
Doctor, consultant/registrar	–	16 (39.1), 16 (39.1)
Clinical nurse specialist	–	9 (22.0)
Rheumatology experience, years		
<5	–	12 (29.3)
6–15	–	15 (36.6)
16–25	–	12 (29.3)
>25	–	2 (4.9)
Involved in EIA clinic, *n* (%)	–	19 (55.9)
Subspecialty interest in RA, *n* (%)	–	29 (76.3)
Involved in research, *n* (%)	23 (21.1)	24 (58.5)
RA patients/week, *n* (%)		
≤10	–	14 (41.2)
11–20	–	16 (47.1)
>20	–	4 (11.8)
Setting where patients are seen, %		
Outpatient department, routine/emergency	–	93, 4
Inpatient	–	6
Patient disease characteristics		
Seropositive, *n* (%)	80 (73.4)	–
Disease duration, years, *n* (%)		–
<3	29 (26.6)	–
3–10	51 (46.8)	–
>10	29 (26.6)	–
DAS-28 at last review, mean (s.d.)	3.6 (1.78)	–
No DAS recorded, *n* (%)	58 (53.2)	–
Smoker, *n* (%)	10 (9.2)	–
≥1 comorbidity, *n* (%)	81 (74.3)	–
DMARDs, *n* (%)		
None	7 (6.4)	–
csDMARD only	61 (56.5)	–
Biologic DMARD total	48 (44.4)	–
Second-line biologic (or more), *n* (%)	14 (12.8)	–

#### Clinicians

A total of 41 clinicians completed the questionnaire. They were predominately female (65%) and 35–54 years of age (65.9%). Most participants were doctors, with clinical nurse specialists representing 22.0% of the cohort. A total of 55.9% were involved in an early inflammatory arthritis (EIA) clinic. Routine outpatient department was overwhelmingly the most common setting for these encounters, with 93.1% of RA patients seen in this way (Table 1).

### Technology use

#### People with RA

**Figure 1.**
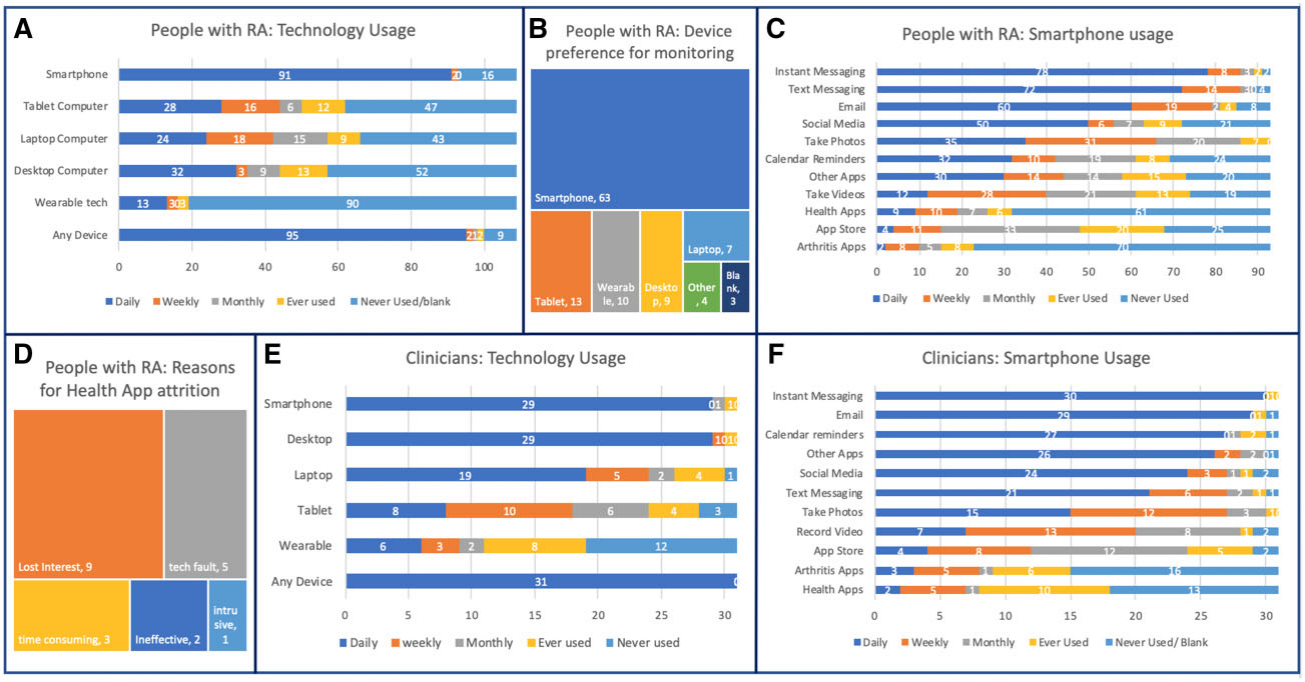
People with RA and clinician technology usage and monitoring device preference. **(A–C)** *N* = 109 people with RA, **(D)** *N* = 20 people with RA, **(E, F)** *N* = 31 clinicians

A total of 87.2% used a device daily and 83.5% used a smartphone; 9/109 (8.3%) did not use any technology on a regular basis (Fig. 1A). The mean age of non-daily smartphone users was 70.7 years (s.d. 13.3); 14.6 years older than the median age of the overall group. Considering a preferred device for remote monitoring, 57.8% selected smartphone, with desktop or laptop computer the next most popular choice at 14.7% (Fig. 1B).

Regular smartphone users (*n* = 93) were surveyed on the use of smartphone functions (Fig. 1C). Instant and text messaging were the most commonly used features, used daily by 83.9% and 77.4%, respectively. E-mail and social media were used daily by more than half of the cohort. Photos were a feature used by all smartphone owners, although less regularly. Notably, arthritis apps were unpopular, with 75.3% having never used one. A total of 19.2% reported they had used, and given up, a health app, and gave free-text answers why, which were coded and grouped (Fig. 1D).

#### Clinicians

All 31 clinicians completing this section used a form of technology daily, with 93.5% using a smartphone. Desktop computers had similarly high usage levels (Fig. 1E). Patterns of smartphone use were similar to that of the patient cohort (Fig. 1F). Arthritis apps were infrequently used, with 9.7% of clinicians reporting daily use; 25.8% used them at least monthly and 54.8% of clinicians reported using apps in clinical practice. However, only 6.4% recommended rheumatology apps to their patients. Apps used by clinicians were all disease activity calculators or information databases, with no patient-facing features.

### Challenges in RA care

#### Participants with RA

**Figure 2.**
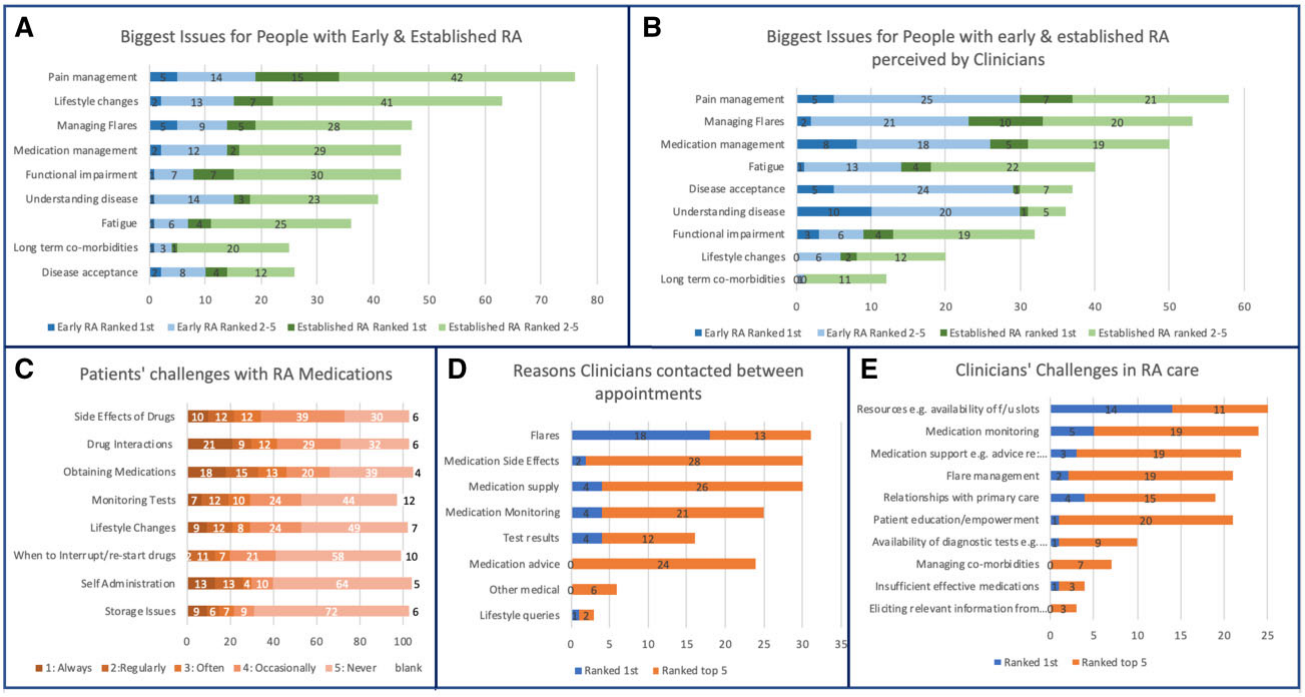
Top five challenges for RA patients and clinicians. Patients and clinicians ranked the top five issues from a list. **(A)** Biggest issues for patients with early RA [within 3 years of diagnosis (*n* = 25)] and established RA [>3 years (*n* = 67)]. **(B)** Biggest issues for patients with early and established RA, perceived by clinicians (*n* = 34). **(C)** Challenges for patients managing RA medications (*n* = 109), rated on a 5-point Likert scale from 1 = always to 5 = never. **(D)** Challenges for clinicians delivering RA care (ranking the top five issues) (*n* = 31). **(E)** Reasons clinicians are most commonly contacted by patients between clinic appointments (ranking the top five issues) (*n* = 33)

A total of 92/109 participants completed this question; 25 with early (<3 years duration) and 67 with established RA. Overall, pain management, lifestyle changes and flare management were ranked as the top three most important issues, with concordance between early and established patients (Fig. 2A). Considering managing conventional synthetic DMARDs (csDMARDs) and advanced therapies, the most commonly identified challenges were interactions (40.7%), obtaining medications (43.8%) and side effects (33%), rated as occurring ‘often’ or more frequently. Storage issues and self-administration were least challenging, with 69.9% and 61.4% selecting ‘never’, respectively. When asked about their greatest concern when commencing a new treatment, participants most commonly selected side effects (50%), followed by obtaining medications (17.2%) and drug interactions (12.5%) (Fig. 2B). A total of 44/96 (45.8%) respondents reported no issues with drug monitoring. The most commonly encountered issue was knowing how regularly blood monitoring was required (29.2%), followed by difficulty attending for blood tests (27.1%).

#### Clinicians

Clinicians ranked disease understanding and acceptance as most important for people with early RA, although these were jointly lowest ranked for those with established disease. Medication management was the third ranked issue for both cohorts. Pain management and flares were the highest ranked issues for people with established RA (Fig. 2C). Of the issues clinicians were most frequently contacted about between appointments, flares were ranked first by 52.9% (88.2% ranked them within the top three most common issues), with queries about medication side effects, medication supply and monitoring tests ranked second, third and fourth, respectively (Fig. 2D).

Considering existing challenges in RA care, resources, e.g. clinic slot availability, emerged as the greatest challenge by a substantial margin (45.2% ranked it first and 80.6% ranked it in the top three). A further cluster of five issues were highly ranked: medication monitoring and support, flares, relationships with primary care and patient education/empowerment. Clinical and communication issues were ranked the least important on average, including supply of effective medications, eliciting relevant information and managing comorbidities (Fig. 2E).

### Clinicians’ attitudes towards disease activity assessment

**Figure 3.**
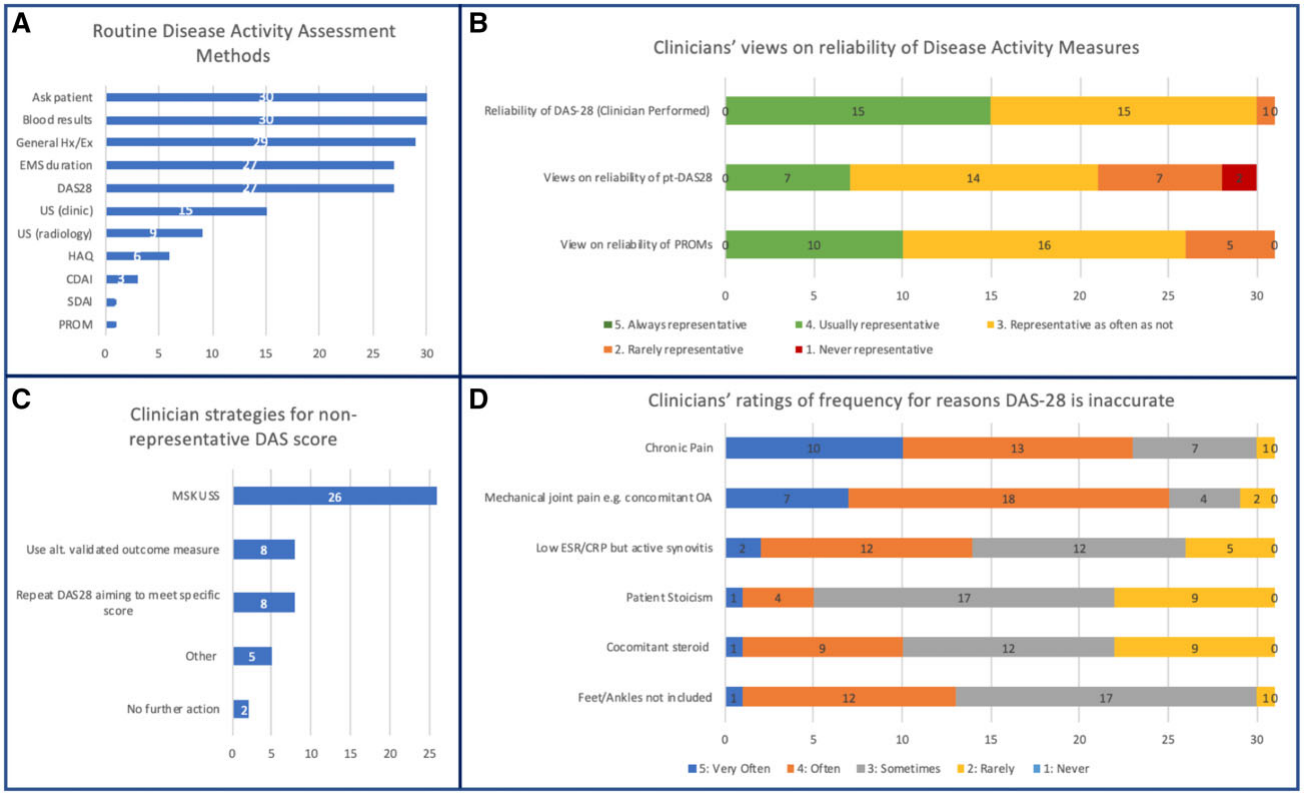
Clinicians’ attitudes towards disease activity assessment. **(A)** Methods used by clinicians to routinely assess disease activity. **(B)** Clinicians’ ratings of reliability of clinician DAS-28, patient DAS-28 and patient-recorded outcome measures (5-point Likert scale rating). **(C)** Clinicians’ ratings of frequency for reasons DAS-28 may be inaccurate (5-point Likert scale rating). **(D)** Clinicians’ strategies when DAS-28 is not in line with clinical assessment

A total of 31 clinicians completed this section. More than 90% of clinicians routinely asked the patient about their general well-being, reviewed blood test results and performed a general history and examination. DAS-28 and morning stiffness duration were used by 87.1%. Clinic-based ultrasound was used by almost half of surveyed clinicians (48.4%). Other validated disease outcome measures were rarely used (Fig. 3A).

Rating perceptions of DAS-28 reliability (Fig. 3B), no clinician rated it ‘always representative’ of disease activity, 48.4% rated it to be ‘usually representative’ and 48.4% rated it neutrally. Chronic pain and mechanical joint pain were the most common contributors to non-representative DAS-28 scores (Fig. 3C). The perceived reliability of patient-reported outcome measures and patient-reported swollen and tender joint counts were rated. Patient-reported joint counts were perceived to be less reliable than patient-reported outcome measures, with 29% scoring patient-reported joint counts ‘rarely’ or ‘never’ representative of true disease activity (Fig. 3B).

Clinicians’ strategies when DAS-28 did not align with their clinical assessment were most commonly ultrasound (83.9%), although 25.8% reported repeating the DAS-28, aiming to meet a specific score (Fig. 3D).

### Useful services provided by technology

**Figure 4.**
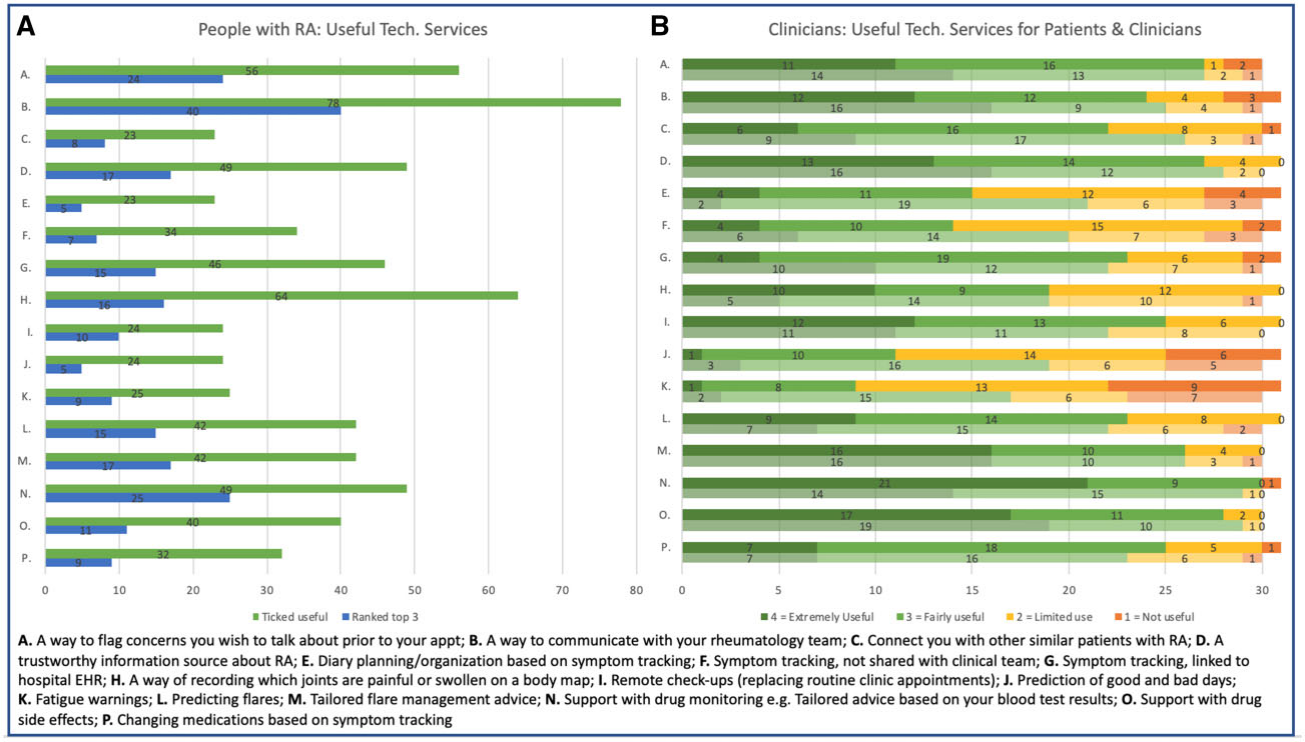
People with RA and clinicians ratings how useful prospective services provided by technology would be. **(A)** Ratings from people with RA of useful services, ranking the top three most useful services and any services they regarded as useful to them (*n* = 109). **(B)** Clinician (*n* = 31) ratings on a 4-point Likert scale of how useful they thought each service would be to them (top) and their patients (bottom)

Considering how useful potential technology services might be (Fig. 4A), 83/109 patients ranked the features most useful to them (24 patients ticked but did not rank features, 7 did not respond). ‘A way to communicate with the rheumatology team’ was top ranked by 72.8%, with 48.8% ranking in their top three. The second top-ranked feature was ‘support with drug monitoring’, with 30.1% ranking this in the top three, and was selected by 48% of participants as ‘useful’, the fourth ranked most useful feature alongside ‘a trustworthy information source about RA’. ‘A way to flag concerns you wish to discuss with your clinical team prior to your appointment’ was the third most selected feature, selected by 52.3% (28.9% ranked top three). ‘A way of recording which joints are painful and swollen on a body map’ was rated ‘useful’ by 59.8%, but <20% ranked it within their top three. ‘Remote check-ups (replacing routine clinical appointments)’ was considered useful by only 22.4% of patients. Fewer than 30% of patients felt that technology changing their medication based on their symptoms would be useful.

A total of 31 clinicians rated the same potential technology services. They considered both their use to clinicians, and to their patient group, with congruence of the top five most positively rated features in both groups (Fig. 4B):

1. Support with drug monitoring based on recent blood tests (96.8% extremely/fairly useful to clinicians; 96.7% to patients)
2. Support with managing drug side effects (93.3% extremely/fairly useful to clinicians; 96.7% to patients)
3. A trustworthy RA information source (87.1% extremely/fairly useful to clinicians; 93.3% to patients)
4. A way for patients to flag concerns they wish to discuss prior to their appointment (87.1% extremely/fairly useful to clinicians; 93.3% to patients)
5. Tailored flare management advice, based on symptom tracking (90.0% extremely/fairly useful to clinicians and patients)

Four features received more negative than positive responses in terms of their use to clinicians:

1. Fatigue warnings, based on symptom tracking input (71% rated limited/no use)
2. Prediction of good and bad days based on symptom tracking input (64.5% rated limited/no use)
3. Symptom tracking, not shared with clinical care team (54.8% rated limited/no use)
4. Helping patients to organize and make decisions about future daily activity based on their symptoms (51.6% rated limited/no use)

However, there was a greater discrepancy between how clinicians rated what they considered useful to them and what might be useful to patients. When rating ‘useful to patients’, no features received a net negative response.

### Barriers to the use of technology to support care

**Figure 5.**
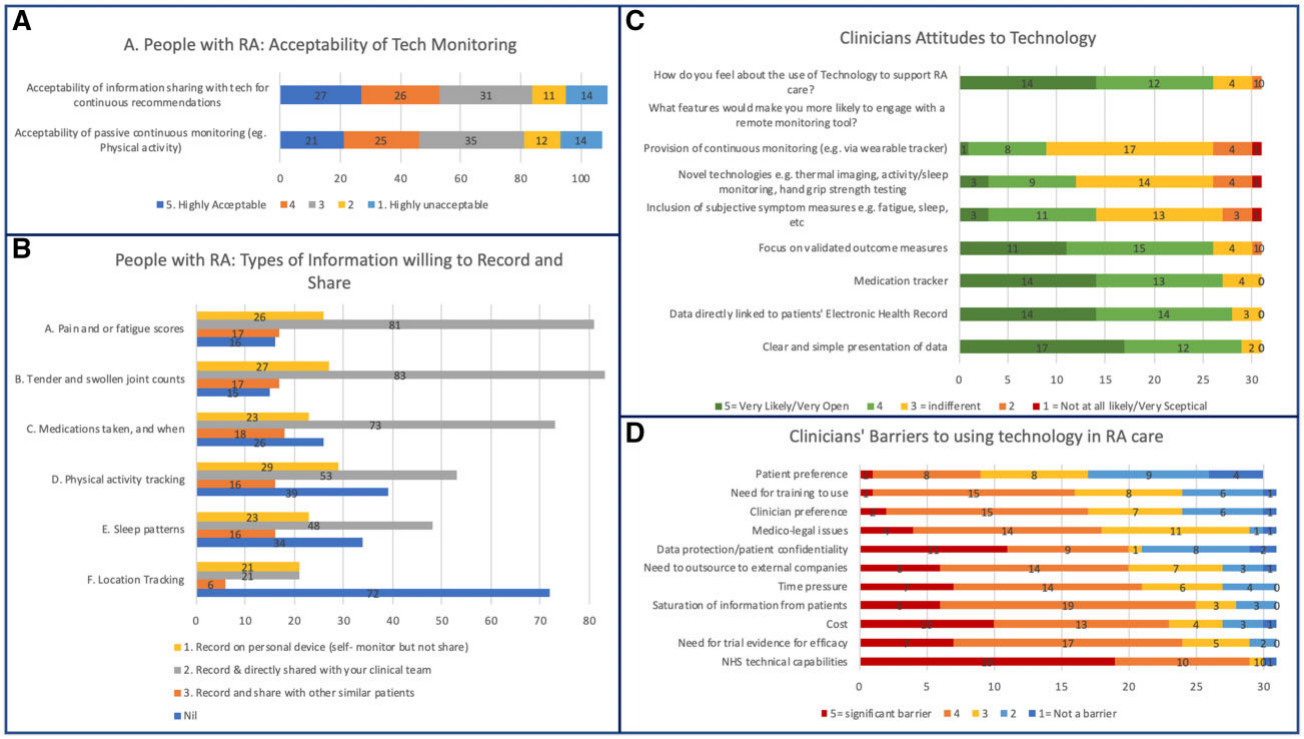
Perceived barriers to technology. **(A)** Acceptability of using technology to monitor disease to people with RA (*n* = 109). **(B)** Types of information people with RA are willing to record and share (*n* = 109). **(C)** Clinicians’ attitudes towards technology and features most likely to promote clinician engagement (*n* = 31). **(D)** Perceived barriers by clinicians to using technology in RA care (*n* = 31)

Rating preferences for recording and sharing data with an mHealth monitoring tool (Fig. 5A), 53.2% of participants living with RA chose ‘monthly’. Only 8.3% selected ‘not at all’ and 12.8% were willing to provide data daily or more frequently. A total of 74.3% gave positive or neutral responses when rating the acceptability of continuous monitoring. When asked about preparedness to track symptoms/activities to receive continuous recommendations, 48.6% selected acceptable or highly acceptable, 28.4% neutral and 22.9% unacceptable/highly unacceptable. Participants were further asked what information they would be prepared to record and/or share, and with whom (Fig. 5B). For all options, sharing information directly with the clinical team was the most popular and sharing information with other patients the least. Recording information on a personal device but not sharing it was only marginally more acceptable than sharing with other patients. Participants were most willing to share clinical information, such as pain scores, joint counts or medication tracking, rather than lifestyle information (e.g. physical activity tracking, sleep patterns or location tracking); 8.7% of patients left all options blank, suggesting they would be unprepared to record or share any of the proposed information.

A total of 31 clinicians answered three questions explicitly addressing attitudes towards utilizing novel technology within RA care (Fig. 5C). Responses to ‘How do you feel about the use of technology to support RA care?’ were largely positive, with 83.9% selecting ‘very open’ or ‘somewhat open’. Rating whether specific features would make them more likely to engage with a remote monitoring tool, three features received no negative responses, with all clinicians rating these as ‘very likely’ or ‘likely’ to encourage them to engage: clear and simple presentation of data, direct linkage of data to patients’ electronic health record and medication tracking. ‘Provision of continuous monitoring’ was least positively scored (80% answering negatively or indifferently). ‘Novel technologies’ and ‘subjective measures’ were similarly scored (61.3% and 54.8% rating either negatively or neutrally). The greatest barrier to the use of technology to support care identified by clinicians was ‘NHS technical capabilities’ (93.5%). Only ‘patient preference’ was rated as either ‘not a barrier’ or neutral, with 41.9% scoring ‘not a barrier’.

## Discussion

This study assessed current technology usage and attitudes of people with RA and their clinicians towards utilising technology to support care and the perceived opportunities and barriers to this. It additionally explored the existing challenges of living with and managing RA in order to draw out potential opportunities for technology not explicitly identified by end users. Our study is consistent with existing published work in European cohorts [19, 20], finding that while people with RA have high levels of smartphone usage, and are largely eager to use mHealth, uptake of existing technologies is low, with only 13.8% using an arthritis app regularly. Our study extends this finding to clinicians, as while more than half our cohort reported using apps in clinical practice, these were all disease activity calculators or information resources, with only 2/31 recommending rheumatology apps to patients. This is congruent with a review of existing commercially available apps, which found that RA apps fall into two categories: simple calculators for rheumatologists and data tracking tools for people with RA [26]. Thus there is a clear appetite for mHealth solutions, as yet unmet by existing technologies.

We found existing challenges for people living with RA to be pain management, lifestyle changes, medication management and flares. The substantially higher ranking of ‘lifestyle changes’, ‘pain management’ and ‘functional impairment’ by people with RA suggest clinicians underestimate the impact that RA has on their patients. These areas present an unmet need and thus an opportunity for mHealth to provide support for people managing day-to-day life with RA, which may help increase engagement beyond simple remote monitoring. People with RA did not rank disease acceptance and understanding nearly so highly as their clinicians, perhaps because these are not conscious processes, but nevertheless, the high ranking by rheumatologists suggests an area of potential growth for mHealth solutions, providing accessible education resources and support solutions tailored to users’ demographics. Clinicians primarily focus on aspects of disease that are directly modifiable by them, within the time-limited constraints of f2f appointments, such as flares and medications. Insufficient resources are the greatest existing challenge for clinicians, strengthening the need for mHealth to reduce the pressure on f2f follow-up slots.

This questionnaire was conducted just prior to the onset of the COVID-19 pandemic, so these challenges will have only deepened with the backlog of patients produced by the pandemic [27].

Challenges in DMARD prescribing is a significant burden to both patients and clinicians, with patients struggling to obtain medications and concerns about side effects and interactions. Medication queries are the most frequent reasons for contact with clinicians between planned reviews. Improving existing pathways for DMARD prescribing and monitoring, with side-effect support, is thus a clear opportunity for mHealth technology to improve RA care and reduce the burden on rheumatology services.

Clinical assessment of RA activity is multifaceted due to the lack of a reliable biomarker to track disease activity. While the DAS-28 is the most commonly used composite measure, most surveyed clinicians considered it to be a flawed measure with many potential confounders, e.g. fibromyalgia. Any remote monitoring measure will need to be multifaceted in order to accurately assess disease activity. Over time, data collected from mHealth monitoring, corroborated clinically, may facilitate the development of novel disease activity measures, e.g. in the ActConnect study, where machine learning methods were used to predict flares based on activity tracking in people with RA and axial spondyloarthropathies [28]. Considering opportunities, people living with RA rated access to their rheumatologist as the most attractive opportunity for technology by a substantial margin, although this was less highly ranked by clinicians, likely due to concerns about the potential for improved accessibility to create an additional workstream, given 80.6% of surveyed clinicians rated ‘saturation of information from patients’ as a barrier to using technology. A way to flag concerns prior to appointments, support with drug monitoring and side effects and providing trustworthy information were highly rated by both groups.

People with RA are generally willing to share information about their disease with their clinical team, with a general preference for sharing data rather than tracking on their individual device. Clinicians are open to using technology within the care pathway but consider NHS technical capabilities, need for trial evidence of efficacy, cost and time as barriers, in addition to concerns regarding mHealth generating data overwhelm.

The strengths of this study are its cross-disciplinary design, with input from both computer scientists and rheumatologists ensuring both clinical and technical aspects were considered throughout the process. Consistent input from RA patient groups ensured the target group was placed first and foremost. Our questionnaire was distributed on paper, eliminating the bias of requiring technology literacy to participate, ensuring a broad spectrum of views. Exploring broader aspects of living with RA and existing challenges in care enabled us to extract information on potential opportunities and barriers beyond those explicitly identified by participants. A possible criticism of this questionnaire study is that many of the issues addressed might be better addressed by qualitative methods. However, our questionnaire forms part of a mixed-methods study, with further results to follow from qualitative analysis of semi-structured interviews with people with RA and clinicians, which explores the themes identified in greater depth and will allow us to triangulate data from both sides to strengthen drawn conclusions.

Potential limitations of the study are its single-centre design and convenience sampling method. However, a diverse population of people with RA were surveyed, in terms of ethnicity, age and disease duration. The clinician arm of the study was multicentre and demonstrated that similar issues were experienced in a variety of centres across London, although this may generalize less well to non-urban settings. The exclusion of primary care physicians and RA patient caregivers, as recommended in EULAR’s points to consider for remote care [24] is a limitation, and an area for further work. Finally, methodological discrepancies between the clinician and patient questionnaires prevented like-for-like comparisons of potential technology services.

## Conclusions

In spite of good levels of technology literacy and willingness from both sides, our cohort did not use technology to support care, suggesting that existing available software is either poorly publicized or inadequate, either in function or user experience. Access, medication support and disease education are mutually agreed opportunities for future mHealth technologies. Existing challenges in the healthcare system, e.g. limited resources, and the need for integration with existing technical systems were the greatest barriers identified. The qualitative arm of this mixed-methods study, in which people with RA and clinicians completed semi-structured interviews, will explore these findings in more granular depth and detail.

## Supplementary Material

rkad089_Supplementary_Data

## Data Availability

The data underlying this article will be shared upon reasonable request to the corresponding author.
