# Supplementary material for: Attitudes towards technology supported rheumatoid arthritis care: investigating patient- and clinician-perceived opportunities and barriers
Source: Rheumatol Adv Pract. 2023 Oct 26;7(3):rkad089. doi: 10.1093/rap/rkad089 (PMC10684358; doi:10.1093/rap/rkad089)
Supplement: rkad089_Supplementary_Data [file rkad089_supplementary_data.zip › AtTRA Clinician Questionnaire final.docx]

**AtTRA: Attitudes to Technology Supported Rheumatoid Arthritis Care**

**Clinician Questionnaire**

**Principal Investigator: Dr. Frances Humby**

**IRAS ID number: 264690**

***Please note: These questions will be loaded as described to SurveyMonkey™ as an online questionnaire***

**Demographics**

- Age (brackets - decades)
  - How old are you? (Tick one box only) 18-24 25-34 35-44 45-54 55-64 65-74 75-84 85 and over
- Gender (What is your gender? [male, female, other (please specify)]
- Grade
  - Rheum SpR (>1 year experience)
  - Rheum Consultant
    - Subspeciality *[Tick all that apply: Rheumatoid Arthritis; Psoriatic Arthritis; Spondyloarthropathy; SLE/CTDs; Autoinflammatory diseases; Gout; Sports medicine; Adolescent Rheumatology; MSK Ultrasound; Non-Inflammatory e.g. OA, CTS etc; Metabolic Bone disease; Other (please specify)]*
  - Rheum Clinical Nurse Specialist or Allied Health Professional (>1 year experience)
    - Subspecialty
  - Years Worked within speciality *[Brackets: <3, 3-5, 6-10, 11-15, 16-20, 21-25, >25]*
  - Are you involved in clinical research? *[Y/N]*
    - If so, what is your area of research *[free text]*

**Understanding Daily Life**

1. How many RA patients do you see on average/week

*[brackets <=5, 6-10, 11-15 16-20, >20]*

*In what setting do you see those patients? Please mark percentages (must add up to 100%)*

- 1. Inpatients *%*
  2. Emergency/Ambulatory Care/Hot clinic *%*
  3. Routine outpatient *%*

1. Involvement in Early Arthritis Clinic? *Y/N*
2. What are the issues that you are most frequently contacted about by Rheumatoid Arthritis patients **between** appointments?

*[Please rank from most common to least common:]*

- 1. Flares;
  2. medication side effects;
  3. medication monitoring tests;
  4. supply of medications;
  5. medication advice e.g. intercurrent infections, surgery etc;
  6. requesting test results;
  7. other medical issues;
  8. lifestyle queries;
  9. other(please specify)

**Understanding patients**

1. What do you think the biggest issues are for patients with Rheumatoid Arthritis?

*[Rank 5 most important: stratify- early diagnosis vs. established RA]*

- 1. disease acceptance,
  2. understanding the disease;
  3. managing flares;
  4. pain management;
  5. medication management;
  6. lifestyle changes;
  7. functional impairment/disability;
  8. long term comorbidities e.g. cardiovascular health, osteoporosis etc.
  9. fatigue
  10. other (please specify)

**Understanding the disease**

1. What methods do you use routinely in clinic to assess patient disease activity? [tick all that apply]
   1. Ask the patient
   2. General history and examination
   3. Duration of Morning Stiffness
   4. DAS28
   5. CDAI
   6. SDAI
   7. HAQ score
   8. Patient Reported Outcome Measure e.g. RAPID3, RADAI5 (please specify)
   9. Ultrasound (done in clinic)
   10. Ultrasound (requested in radiology dept)
   11. Blood test results
   12. Any other methods (please specify)
2. In considering an ideal tool for assessment of Rheumatoid Arthritis Activity, what would be the most important aspects of such a hypothetical monitoring tool? [Please rank most important-least important]
   1. Patient acceptability
   2. Accuracy
   3. Speed of assessment
   4. Simplicity
   5. Proven Validity within clinical trial
   6. Sensitivity to change
   7. Depth of information
   8. Objectivity
   9. Cost
   10. Impact on clinical decision making
   11. Other (please specify)
3. Does the DAS-28 score usually accurately reflect patients disease activity?

*[Likert scale 1= never representative 5= Always representative]*

- 1. What are the commonest reasons that a patient has a non-representative DAS score *[please rank from most common 1 to 6 least common]*
     1. Chronic Pain Syndrome
     2. Mechanical Joint pain e.g. concomitant OA
     3. Patient Stoicism
     4. Feet/Ankles not included
     5. Concomitant steroid therapy
     6. Patients with persistently low inflammatory markers in spite of active synovitis
     7. Other (please specify)
  2. If a patient’s DAS score is not in line with your clinical assessment, how do you address this? *[Tick all that apply]*
     1. General clinical assessment
     2. MSK Ultrasound Scan
     3. Use alternate validated outcome measure
     4. Repeat DAS28 with aim to meet specific score
     5. No further action taken
     6. Other (please specify)
  3. What are your views on the reliability of patient self-reported tender and swollen joint counts?

*[Likert Scale: 1= not at all reliable 5= completely reliable]*

*If score <5 please elaborate on your reservations… Free text*

- 1. What are your views on the reliability of patient reported outcome measures as a tool for disease activity monitoring ?

*[Likert Scale: 1= not at all reliable 5= completely reliable]*

- 1. Please provide any further comments regarding DAS28 as an assessment tool below *[Free text]*

1. What are the biggest challenges in RA care and monitoring? *[Please rank 1 = biggest challenge to 7= least challenge]*
   1. Medication support for patients e.g. advice for intercurrent infections, surgery, etc
   2. Medication monitoring
   3. Patient education/empowerment
   4. Resources e.g. availability of follow-up appointments,
   5. Availability of diagnostic tests e.g. ultrasound
   6. Eliciting relevant information from patients
   7. Flare management
   8. Relationships with primary care
   9. Insufficient effective medications
   10. Managing comorbidities
   11. Other (please specify)
2. What are the most frustrating aspects of RA patient care? [Free text]

**Attitudes to Technology**

1. How do you feel about the use of technology to support Rheumatoid Arthritis care? *[Likert scale 1= very sceptical 2= somewhat sceptical 3= indifferent 4= somewhat open 5= very open]*
2. Do you use any apps in your clinical practice? E.g. DAS calculators, *[YES / NO… If yes, please specify {free text}]*
   1. If so, how regularly do you use these?
   2. What features of these apps are useful? *please specify {free text}]*
3. Do you recommend any Rheumatology Apps to patients? [*Y/N: if yes, Please specify:*]
   1. If so, what features of this app do you think are useful to patients?

**Opportunities of technology**

1. What services provided by technology would be useful to you/your patient group  *[4- point Likert scale 1= not useful 2= limited use 3= fairly useful 4= extremely useful]*
   1. Providing a way for patients to flag up concerns they wish to talk about with the clinical team prior to their appointment (e.g. medication side effects, pregnancy planning, etc.)
   2. As a way for patients to communicate with the rheumatology team
   3. As a way for patients to communicate with other similar patients with rheumatoid arthritis
   4. As a general trustworthy information resource about rheumatoid arthritis
   5. Helping patients to organise and make decisions about future daily activity based on their symptoms
   6. Symptom Tracking on patients’ personal devices (not shared with clinical care team)
   7. Symptom Tracking on patients’ personal devices (with information directly linked to their hospital electronic health record)
   8. A way of recording which joints are painful or swollen on a diagram
   9. Remote check-ups (e.g. replacing routine clinic appointments)
   10. Prediction of good days and bad days
   11. Giving warnings for patients that they are becoming or will soon be fatigued
   12. Flare prediction (based on symptom tracking input)
   13. Providing patients with tailored advice about how to manage flares (based on symptom tracking input)
   14. Support for patients with drug monitoring e.g. direct advice based on their most recent blood tests
   15. Support for patients with drug side effects and how to manage them
   16. Up-titrating or down-titrating patients medications based on their symptom tracking inputs
   17. Other (please state)
2. What features would make you more likely to engage with a remote monitoring tool? [Likert scale 1 = not at all likely 5= very likely]
   1. Data directly linked to patients’ EHR
   2. Clear and simple presentation of data
   3. Provision of continuous monitoring
   4. Focus on validated outcome measures
   5. Inclusion of subjective symptom measures e.g. fatigue, sleep etc.
   6. Medication tracker
   7. Use of novel technologies such as:
      1. Thermal imaging (e.g. to detect joint inflammation)
      2. Activity monitoring
      3. Sleep monitoring
      4. Hand grip strength testing
      5. Mobile ultrasound scanning
      6. Other novel technology (please state)
   8. Other features (please state)
3. Are there any other ways in which you think technology could be used to improve care for patients with Rheumatoid Arthritis? *[Free text]*

**Barriers to technology**

1. What are the barriers to using technology to support RA care? [Likert scale 1= not a concern 5= severe concern]
   1. Data protection/patient confidentiality
   2. NHS technical capabilities
   3. Medico-legal
   4. Patient preference
   5. Clinician preference
   6. Need for trial evidence for efficacy
   7. Cost
   8. Saturation of information from patients
   9. Time pressure
   10. Need to outsource to external companies
   11. Need for training to use
   12. Other (please state)
2. Please elaborate on any specific concerns you have regarding technology supported RA care [Free text]

**Current Technology Usage**

Please indicate the extent you currently use each of the following kinds of devices:

(5 part Likert scale : daily, weekly, monthly, have used ever, never used)

- Desktop computer
- Laptop computer
- Tablet computer
- Smartphone
- Wearable tech tracker (specify)
- Other (specify)

**Smartphone Usage**

**(If you own a smartphone)**

If you own a smartphone, please indicate the extent you currently use it for each of the following:

(5 part Likert scale : daily weekly monthly, have used ever, never used)

- Send or receive SMS
- Instant messaging
- Send or receive emails
- Calendar reminders
- Take Photos
- Record Video
- Use social media
- Use arthritis apps
- Use any other mHealth apps
- Use of an app store to find and download new apps
- Use other apps
