# Supplementary material for: Attitudes towards technology supported rheumatoid arthritis care: investigating patient- and clinician-perceived opportunities and barriers
Source: Rheumatol Adv Pract. 2023 Oct 26;7(3):rkad089. doi: 10.1093/rap/rkad089 (PMC10684358; doi:10.1093/rap/rkad089)
Supplement: rkad089_Supplementary_Data [file rkad089_supplementary_data.zip › AtTRA Patient Questionnaire v1.4.docx]

**AtTRA: Attitudes to Technology supported Rheumatoid Arthritis Care**

**Section 1: Patient Demographic Information**

1. Education level: What is the highest education qualification you have been awarded? (Please tick one)
   - Postgraduate degree or equivalent
   - Degree or equivalent
   - Higher education
   - A Level or equivalent
   - GCSE grades A*-C or equivalent
   - Other qualifications
   - No qualification
   - Don’t know
2. Employment status:
   1. Are you currently working?
      - Yes: Full time employment
      - Yes: Less than full time/Flexible employment
      - Yes: Freelance
      - No: Unemployed
      - No: Retired
      - Other
   2. If you are currently working, please tell us your job

________________________

- 1. If you are not currently working, is this due to ill health?

YES NO

**Section 2: Current Technology Usage**

1. Please indicate the extent you currently use each of the following kinds of devices: (please circle one)

- **Desktop computer**

1 2 3 4 5

Daily Weekly Monthly Ever Used Never Used

- **Laptop computer**

1 2 3 4 5

Daily Weekly Monthly Ever Used Never Used

- **Tablet computer**

1 2 3 4 5

Daily Weekly Monthly Ever Used Never Used

- **Smartphone**

1 2 3 4 5

Daily Weekly Monthly Ever Used Never Used

- **Wearable tech tracker (specify)**

1 2 3 4 5

Daily Weekly Monthly Ever Used Never Used

Specify what type if used:

- **Other (specify)_________________________________________________**

1 2 3 4 5

Daily Weekly Monthly Ever Used Never Used

2. Of the following, which would you be **most** willing to use to help monitor your arthritis? [TICK ONE]

- Desktop computer
- Laptop computer
- Tablet computer
- Smartphone
- Wearable tech tracker (specify)
- Other (specify)

3. Of the following which would you be **least** willing to use to help monitor your arthritis?  [TICK ONE]

- Desktop computer
- Laptop computer
- Tablet computer
- Smartphone
- Wearable tech tracker (specify)
- Other (specify)

**Smartphone Usage**

**(If you own a smartphone)**

1. If you own a smartphone, please indicate the extent you currently use it for each of the following:

- **Send or receive sms (text messaging)**

Daily Weekly Monthly Ever Used Never used

- **Instant messaging e.g. Whatsapp**

Daily Weekly Monthly Ever Used Never used

- **Send or receive emails**

Daily Weekly Monthly Ever Used Never used

- **Calendar reminders**

Daily Weekly Monthly Ever Used Never used

- **Take Photos**

Daily Weekly Monthly Ever Used Never used

- **Record Video**

Daily Weekly Monthly Ever Used Never used

- **Use social media**

Daily Weekly Monthly Ever Used Never used

- **Use arthritis apps**

Daily Weekly Monthly Ever Used Never used

- **Use any other mHealth apps**

Daily Weekly Monthly Ever Used Never used

- **Use of an app store to find and download new apps**

Daily Weekly Monthly Ever Used Never used

- **Use other apps**

Daily Weekly Monthly Ever Used Never used

2. If you have ever used a health-related software, mobile health app or wearable tracker and then given up use, please indicate why. *[Tick all that apply]*

- - Not applicable (never used them)
  - Ineffective
  - Lost interest
  - Broken/Hardware fault
  - Too time consuming
  - Too intrusive
  - Reminder of failure
  - Cost (e.g. subscription fee)
  - Other (please state)

**Section 3: Understanding Disease**

**Understanding the disease in daily life**

1. To what extent has your arthritis changed your daily life? *[Please circle the number which most closely represents your symptoms]*

**1 2 3 4 5**

Not at all Somewhat Severely

1. What do you think the biggest issues are for patients with Rheumatoid Arthritis? *[Rank the 5 most important to you, with ‘1’ representing the most important]*
   - disease acceptance
   - understanding the disease
   - managing flares
   - pain management
   - medication management
   - lifestyle changes
   - functional impairment/disability
   - long term comorbidities e.g. cardiovascular health, osteoporosis etc.
   - fatigue
   - other (please specify)
2. To what extent does fatigue play a role in your life? *[Please circle the number which most closely represents your symptoms]*

**1 2 3 4 5**

Not at all Somewhat Severely

**Understanding flares:**

1. What are the symptoms you associate with having a flare? *[Tick all that apply]*
   - Joint pain
   - Pain (other than joints)
   - Joint swelling
   - Stiffness
   - Fatigue
   - Sleep disturbance
   - Low mood
   - Reduced mobility
   - Loss of independence
   - Reduced mental power (e.g. “Brain Fog”)
   - Other (please state)
2. How regularly, if ever, do you experience flares of your arthritis?

*[Please circle the number which most closely represents your symptoms]*

**1 2 3 4 5**

Extremely rarely Infrequently Monthly Weekly Continuously

1. How long do your flares usually last? *[Please tick the box which most closely represents your symptoms]*
   1. What is the **longest** they last?
      - <1day
      - 1-3 days
      - 3-7 days
      - 8-14 days
      - >14 days
   2. What is the **shortest** they last?
      - <1day
      - 1-3 days
      - 3-7 days
      - 8-14 days
      - >14 days
   3. How long do they **commonly** last?
      - <1day
      - 1-3 days
      - 3-7 days
      - 8-14 days
      - >14 days
2. What do you do to self-manage flares when they arise?

*Please tick all that apply, then rank from 1-3 the most beneficial to you personally*

| **Strategy** | **Tick all that apply** | **Please rank the three most beneficial to you personally** |
| --- | --- | --- |
| a. Simple painkillers |  |  |
| b. Topical treatments (e.g. creams, gels etc) |  |  |
| c. Prescribed painkillers |  |  |
| d. Steroid tablets (increase dose or start course without seeking medical advice) |  |  |
| e. Herbal remedies/supplements |  |  |
| f. Alternative therapies e.g. homeopathy, acupuncture, reiki etc. |  |  |
| g. Rest |  |  |
| h. Time off work |  |  |
| i. Exercise |  |  |
| j. Massage |  |  |
| k. information seeking e.g. internet research |  |  |
| l. Social/emotional support |  |  |
| m. Other (please state) |  |  |

1. How do you access care between hospital appointments if you are experiencing a flare? *[tick all that apply]*
   - GP;
   - Urgent Care/A&E;
   - Call Consultant’s secretary;
   - Call Rheumatology Nurse;
   - Call Rheumatology Department
   - Other (please state)
2. How easy or difficult is it to contact your rheumatology nurse or doctor if needed? *[Please circle the number which most closely represents your experience]*

**1 2 3 4 5**

Very Easy Very Difficult

1. What, if any, problems do you encounter when trying to contact your rheumatology nurse or doctor? *[Please tick all that apply]*
   - Not applicable (never have problems)
   - Don’t know contact information
   - Long wait to get through
   - Passed from one person to another
   - Told someone would call back but they didn’t
   - Other (please state)
2. When you do reach them, what is usually the outcome? *[Please tick all that apply]:*
   - Not applicable (never reached anyone),
   - Urgent appointment given;
   - telephone advice given;
   - prescription dispensed e.g. for steroid tablets/injection;
   - told to attend GP practice;
   - advised on self-management e.g. painkillers;
   - further tests booked e.g. ultrasound scan/bloods;
   - advised to increase dose of usual tablets;
   - flare has improved by time we I reach department;
   - Other (please state)

**Understanding pain:**

1. How often do you experience pain (outside of a ‘flare up’)

*[Please circle the number which most closely represents your experience]*

**1 2 3 4 5**

Constantly Daily Weekly Infrequently Never

1. What do you do to self-manage pain (outside of your flares)?

*Tick all that apply, then rank from 1-3 the most beneficial to your personally*

| **Strategy** | **Tick all that apply** | **Please rank the three most beneficial to you personally** |
| --- | --- | --- |
| a. Simple painkillers |  |  |
| b. Topical treatments (e.g. creams, gels etc) |  |  |
| c. Prescribed painkillers |  |  |
| d. Steroid tablets (increase dose or start course without seeking medical advice) |  |  |
| e. Herbal remedies/supplements |  |  |
| f. Alternative therapies e.g. homeopathy, acupuncture, reiki, |  |  |
| g. Rest |  |  |
| h. Time off work |  |  |
| i. Exercise |  |  |
| j. Massage |  |  |
| k. information seeking e.g. internet research |  |  |
| l. Social/emotional support |  |  |
| m. Other (please state) |  |  |

**Arthritis Medications and Monitoring**

1. To what extent are each of the following challenges with respect to taking medications for your Rheumatoid Arthritis?

*[Please circle the number which most closely represents your experience]*

- 1. Obtaining medications

**1 2 3 4 5**

Always Regularly Often Occasionally Never

- 1. Storage issues

**1 2 3 4 5**

Always Regularly Often Occasionally Never

- 1. Self-administration (eg swallowing tablets, taking injections, etc)

**1 2 3 4 5**

Always Regularly Often Occasionally Never

- 1. Monitoring tests

**1 2 3 4 5**

Always Regularly Often Occasionally Never

- 1. Side effects of drugs

**1 2 3 4 5**

Always Regularly Often Occasionally Never

- 1. Lifestyle changes (e.g. modifying alcohol intake, contraception advice, etc.)

**1 2 3 4 5**

Always Regularly Often Occasionally Never

- 1. Knowing when to interrupt/re-start treatment (e.g. infections, surgery, pregnancy etc.)

**1 2 3 4 5**

Always Regularly Often Occasionally Never

- 1. Knowing if it’s safe to take other medications with your Rheumatoid Arthritis drugs

**1 2 3 4 5**

Always Regularly Often Occasionally Never

- 1. Other (please state)
  2. **2 3 4 5**

Always Regularly Often Occasionally Never

1. What are your biggest concerns when starting a new medication?

*Please score the ONE* ***MOST*** *important and* ***LEAST*** *important to you*

| **MOST important** | **Concerns** | **LEAST important** |
| --- | --- | --- |
|  | a. Obtaining medications |  |
|  | b. Storing medications |  |
|  | c. Self-administration (e.g. swallowing tablets, self-injecting, etc) |  |
|  | d. Monitoring tests |  |
|  | e. Drug side effects |  |
|  | f. Lifestyle changes e.g. modifying alcohol intake, conception advice etc |  |
|  | g. Knowing when to interrupt/re-start treatment (e.g. infections, surgery, pregnancy, etc) |  |
|  | h. Drug interactions i.e. knowing it’s safe to take other medications with your arthritis medications |  |
|  | i. Other (please state) |  |

1. What, if any, problems do you have with drug monitoring for adverse effects (e.g. blood tests, eye monitoring) *Tick all that apply:*
   - None
   - Difficulty attending for blood tests e.g. finding time off work
   - Difficulty obtaining blood test forms
   - Unpleasant experience (e.g. needlephobia)
   - Concern that results aren’t monitored
   - Knowing how regularly monitoring required/ Discrepancies between GP and hospital monitoring
   - Other (please state)

**Stopping/Interrupting Treatment**

1. If you have ever had to interrupt your RA treatment for any reason, what was the reason for stopping? [*Please tick all that apply*]
   - Not applicable (have never interrupted treatment)
   - Advised by rheumatology team:
     - Due to monitoring test results
     - For surgical procedure
     - Due to infection
     - I am not sure of the reason
     - Other (please state)

______________________

- - Advised by GP
    - Due to monitoring test results
    - Other (please state)

______________________

- Other
  - Advised by surgeon for surgical procedure
  - Advised by friend/family/other source
  - Stopped myself due to infection
  - Ran out of supply
  - Pregnancy/trying to conceive/breastfeeding
  - Side effects
  - Other (please specify)

1. If you have ever stopped treatment, what is the longest  you have stopped treatment for?

*[Please circle the number which most closely represents your experience]*

**1 2 3 4 5**

few days few weeks few months indefinitely permanently

1. What were the consequences of stopping treatment in that case? *[Please tick all that apply]*
   - Not applicable (never stopped treatment)
   - No consequences of stopping
   - Flare of Arthritis (Mild/Moderate)
   - Flare of Arthritis (Severe)
   - Resolution of side effects
   - Other (please specify)

____________________________________

**Clinic Appointments**

1. How often do you find all your concerns are addressed in your Rheumatology clinic appointment  (with a Doctor or Nurse)

*[Please circle the number which most closely represents your experience]*

**1 2 3 4 5**

Always Frequently Occasionally Rarely Never

1. What, if anything, do you do to help you to remember the issues you want to bring up at your appointment? *[Tick one]*
   - Paper list
   - Electronic list
   - Think about the in advance but don’t write it down
   - Do not think about it until the appointment
   - Other (please state) _________________
2. How often **do you** attend clinic appointments with a Rheumatology Specialist (Doctor or Nurse) *[Tick one]*
   - Less than annually
   - Once a year
   - Every 6 months
   - Every four months
   - More frequently
   - Other (please state)
3. Ideally, how often **would you like to** attend clinic appointments with a Rheumatology Specialist (Doctor or Nurse) ) *[Tick one]*
   - Less than annually
   - Once a year
   - Every 6 months
   - Every four months
   - More frequently
   - Other (please state)

**Opportunities of Technology**

1. What services provided by technology would be useful to you?

*[In the first column, please tick any services you think would be useful to you. In the 2^nd^ column, of those you have marked as ‘useful’, please rank the top 3 most important features)*

| **Service** | **Useful?** | **Rank the top three features you would find useful** |
| --- | --- | --- |
| A. Giving a way to flag up concerns you wish to talk about with your clinical team prior to your appointment (e.g. medication side effects, pregnancy planning, etc.) |  |  |
| B. As a way to communicate with your rheumatology team |  |  |
| C. As a way to communicate with other similar patients with rheumatoid arthritis |  |  |
| D. As a general trustworthy information source about rheumatoid arthritis |  |  |
| E. Helping you to organise and make decisions about future daily activity |  |  |
| F. Tracking how your symptoms change on your personal device (i.e. not shared with your clinical care team) |  |  |
| G. Tracking how your symptoms change on your personal device, but with information linked to and stored in your hospital electronic health record |  |  |
| H. A way of recording which joints are painful or swollen on a body map/diagram |  |  |
| I. Remote check-ups (e.g. replacing routine clinic appointments) |  |  |
| J. Prediction of good days and bad days |  |  |
| K. Giving warnings that your are becoming or will soon be fatigues |  |  |
| L. Predicting when flares may happen |  |  |
| M. Giving tailored advice about how to manage flares |  |  |
| N. Support with drugs monitoring e.g. tailored advice based on your most recent blood test results |  |  |
| O. Support with drug side effects and how to manage them |  |  |
| P. Changing your prescriptions based on your symptoms |  |  |
| Q. Other |  |  |

**Barriers of Technology**

1. How often would you be willing to provide information/be prompted to give information about your disease?

*[Please circle the number which most closely represents your experience]*

**1 2 3 4 5**

Hourly Daily Weekly Monthly Not at all

How acceptable would it be for an app to continuously monitor information (e.g. regarding physical activity) without disturbing you?

*[Please circle the number which most closely represents your experience]*

**1 2 3 4 5**

Highly Unacceptable Highly Acceptable

1. If there was an app that would track your activities and symptoms would you be willing to share information with it to get continuous recommendations?

*[Please circle the number which most closely represents your experience]*

**1 2 3 4 5**

Highly Unacceptable Highly Acceptable

1. Which of the following information (if it could be automatically sensed by, or manually entered into, a device)  would you be willing to record or share

| **Information** | **A. Record on personal device (i.e. monitored but not shared with anyone else)** | **B. Record & directly shared with your clinical team** | **C. Record and share with other similar patients?** |
| --- | --- | --- | --- |
| **A. Pain and/or fatigue scores** |  |  |  |
| **B. Tender and swollen joint counts** |  |  |  |
| **C. Which medications you have taken, and when** |  |  |  |
| **D. Physical activity tracking** |  |  |  |
| **E. Sleep patterns** |  |  |  |
| **F. Location tracking** |  |  |  |
| **G. Other (please specify)**  **______________________** |  |  |  |

1. Finally, is there anything else important (relating to the topics covered) that you wish to tell us?
